# Concordance of B‐ and T‐cell responses to SARS‐CoV‐2 infection, irrespective of symptoms suggestive of COVID‐19

**DOI:** 10.1002/jmv.28016

**Published:** 2022-07-30

**Authors:** Marc F. Österdahl, Eleni Christakou, Deborah Hart, Ffion Harris, Yasaman Shahrabi, Emily Pollock, Muntaha Wadud, Tim D. Spector, Matthew A. Brown, Jeffrey Seow, Michael H. Malim, Claire J. Steves, Katie J. Doores, Emma L. Duncan, Timothy Tree

**Affiliations:** ^1^ Department of Twin Research & Genetic Epidemiology, School of Life Course Sciences, Faculty of Life Sciences and Medicine King's College London London UK; ^2^ Department of Ageing and Health Guy's and St Thomas' NHS Foundation Trust London UK; ^3^ Department of Immunobiology, School of Immunology & Microbial Sciences King's College London London UK; ^4^ Guy's and St Thomas' NHS Foundation Trust and King's College London NIHR Biomedical Research Centre King's College London London UK; ^5^ Department of Infectious Diseases, School of Immunology & Microbial Sciences King's College London London UK; ^6^ Department of Endocrinology Guy's and St Thomas' NHS Foundation Trust London UK

**Keywords:** antibody, SARS‐CoV‐2, symptom response, T cell

## Abstract

This study assessed T‐cell responses in individuals with and without a positive antibody response to SARS‐CoV‐2, in symptomatic and asymptomatic individuals during the COVID‐19 pandemic. Participants were drawn from the TwinsUK cohort, grouped by (a) presence or absence of COVID‐associated symptoms (S+, S−), logged prospectively through the COVID Symptom Study app, and (b) anti‐IgG Spike and anti‐IgG Nucleocapsid antibodies measured by ELISA (Ab+, Ab−), during the first wave of the UK pandemic. T‐cell helper and regulatory responses after stimulation with SARS‐CoV‐2 peptides were assessed. Thirty‐two participants were included in the final analysis. Fourteen of 15 with IgG Spike antibodies had a T‐cell response to SARS‐CoV‐2‐specific peptides; none of 17 participants without IgG Spike antibodies had a T‐cell response (*χ* ^2^: 28.2, *p* < 0.001). Quantitative T‐cell responses correlated strongly with fold‐change in IgG Spike antibody titer (*ρ* = 0.79, *p* < 0.0001) but not to symptom score (*ρ* = 0.17, *p* = 0.35). Humoral and cellular immune responses to SARS‐CoV‐2 are highly correlated. We found no evidence of cellular immunity suggestive of SARS‐CoV2 infection in individuals with a COVID‐19‐like illness but negative antibodies.

**Abbreviations**
COVID‐19: coronavirus disease 2019
DMSO: dimethyl sulfoxide
ELISA: enzyme‐linked immunosorbent assays
PBMCs: peripheral blood mononuclear cells
REMAS: Research Ethics Management Application System
ROC: receiver operator characteristic
SARS‐CoV‐2: severe acute respiratory syndrome coronavirus 2
ZCS: Zoe COVID Study

## 1 INTRODUCTION

The COVID‐19 pandemic caused by SARS‐CoV‐2 has been catastrophic to human health, causing over 4 million deaths worldwide by July 2021.^1^ Key to controlling SARS‐CoV‐2 spread is the ability to identify accurately individuals with current or past infection, determining quarantine and contact tracing requirements. Current infection with SARS‐CoV‐2 is diagnosed using PCR (detecting viral RNA) or lateral flow antigen testing); prior infection is typically diagnosed by demonstrating a memory immune (typically antibody) response to SARS‐CoV‐2.

The adaptive immune response to SARS‐CoV‐2 comprises humoral and cell‐mediated components. The humoral (or antibody) response is detectable in convalescent sera approximately 2−3 weeks after infection, with an initial IgM response followed within days by an IgG response, including against the Spike protein (also the target of vaccines^2^) and, less specifically, against the Nucleocapsid protein. Additionally, a neutralizing antibody response may be measurable, which assesses the functional capacity of a convalescent serum to inhibit virus infection in vitro.^3^ Complementary to the antibody response is a cellular response driven by T cells, particularly CD4^+^ T helper cells. When stimulated by a pathogen, naïve CD4^+^ cells differentiate into T helper cell subsets which orchestrate the immune response, including supporting pathogen‐specific cytotoxic (CD8^+^) T cells and stimulating B cells to produce a high‐affinity pathogen‐specific antibody response. Other CD4^+^ T cells differentiate into T regulatory cells, which attenuate immune responses to the pathogen.^4^ Following clearance of infection, pathogen‐specific memory T‐cell responses play an important role in protective immunity and are detectable in peripheral blood long term.

Antibody responses have been detected in most individuals after acute COVID‐19.^3^, ^5^ However, some individuals reporting symptoms suggestive of COVID‐19, including individuals reporting prolonged symptom duration suggestive of the Post‐COVID Syndrome (“Long COVID”), do not have detectable antibody responses.^6^ One possible explanation is infection with other respiratory pathogens (such as influenza virus) whose symptom profile overlaps with COVID‐19, particularly early in the pandemic; however, as the pandemic progressed, with the introduction of social distancing and personal protection, the circulation of other respiratory pathogens declined and SARS‐CoV‐2 became the dominant respiratory infection.^7^ Another possibility is false‐negative testing, as thresholds for defining an antibody response as positive or negative reflect a compromise between assay sensitivity and specificity, neither being 100%. In addition, antibody responses to SARS‐CoV‐2 decline over time, also observed with other coronaviruses including SARS‐CoV‐1.^8^ In contrast, T‐cell responses are usually prolonged and, although their frequency may wane with time, can be demonstrable years after initial infection—for example, T‐cell responses following SARS‐CoV‐1 infection are detectable for over 17 years (to date).^9^

Here we assess humoral and cell‐mediated responses to SARS‐CoV‐2, in symptomatic and asymptomatic individuals during the first UK wave of the COVID‐19 pandemic. In particular, we assess whether individuals with symptoms potentially consistent with COVID‐19, but without a detectable antibody response, have demonstrable cell‐mediated immunity.

## 2 MATERIALS AND METHODS

### 2.1 TwinsUK

The TwinsUK cohort is the largest community‐based cohort of adult twins in the United Kingdom, with >14 000 registered individuals (>7000 pairs) assessed longitudinally over nearly 30 years. Their experience of the COVID‐19 pandemic was closely monitored, with 10 230 individuals participating in regular questionnaires about prior symptoms.^10^, ^11^ Of these, 431 individuals participated in a home visit study during May−June 2020 (study protocol, participant demographics, and inclusion/exclusion criteria previously published^6^). Briefly, participants were selected based on: (a) proximity of both twins (within 80 miles of St Thomas' Hospital, Westminster); (b) sufficient symptom reporting in the COVID Symptom Study (discussed below) to enable calculation of a COVID “symptom score”^12^; and (c) availability during the study period. Serum samples were tested for IgG antibody against SARS‐CoV‐2 Spike protein.

Subsequently, individuals were classified into four groups, defined by both symptom score and IgG Spike antibody responses from the initial home visit: symptom‐positive, antibody‐positive; symptom‐positive, antibody‐negative; symptom‐negative, antibody‐positive (i.e., asymptomatic infection); and symptom‐negative, antibody‐negative (i.e., control group). Participants were then revisited, to collect peripheral blood mononuclear cells (PBMCs) and a contemporaneous serum sample for repeat antibody testing.

### 2.2 The Zoe COVID Study (ZCS)

2.2.1

The ZCS was launched jointly on March 24, 2020, by ZOE Limited and academics of King's College London, Massachusetts General Hospital, and Lund and Uppsala Universities, through a smartphone application.^12^ Briefly, on registration, participants provide baseline demographic data and subsequently are prompted daily to report their health status (including being asymptomatic), healthcare access, vaccination, testing, and so forth. Data on key symptoms are combined into a “symptom score” from 0 to 1.0, with a score above 0.5 defining probable COVID‐19 (here, “Symptom‐Positive”). During the first UK wave, this model showed 65% sensitivity and 78% specificity for self‐reported SARS‐CoV‐2 infection (defined by reverse transcription PCR).^12^ Many of the TwinsUK cohort also participate in the ZCS, with the linkage of their data.

### 2.3 Humoral assays

Spike and Nucleocapsid protein were expressed as previously described.^13^ Further details of laboratory processing are included in supplementary methods. For binary classification, we used a fourfold increase above background in both IgG Spike (S) and Nucleocapsid (N) antibody titer to define a case as “Antibody‐Positive,” based on previously established thresholds.^13^ For continuous variable analyses, the fold‐change in IgG Spike titer was used.

### 2.4 Analysis of T‐cell responses

PBMCs were isolated from Li Hep blood by density gradient centrifugation using Lymphoprep (Axis Shield), cryopreserved in CS10 n CryoStor® (Sigma‐Aldrich) and stored in vapor phase liquid nitrogen. Cryopreserved PBMCs were thawed, and viability was assessed by trypan blue exclusion following a resting period. Cells were stimulated with peptide pools, including S1 and S2 domains of SARS‐CoV‐2 spike protein, as well as Matrix (M) and Nucleocapsid (N) proteins. Superantigen Entereotoxin B (SEB) was used as a negative control. We used stored PBMCs from pre‐pandemic healthy controls, performing a receiver operating characteristic (ROC) analysis to define thresholds of responses to SARS‐CoV‐2 peptides. Further details, including the identification of T‐cell subtypes by chemokine receptors, are available in supplementary methods.^14^, ^15^, ^16^

### 2.5 Statistical methods

Participant ascertainment is descriptive. The likelihood ratio from ROC curve analysis was used to define the optimal threshold to differentiate positive versus negative total T‐cell responses, as well as T helper and T regulatory responses individually. Associations between binary thresholds of IgG Spike antibody status and combined T‐cell responses were assessed with *χ* ^2^ testing. When analyzed as continuous variables, antibody response, symptom score, and T‐cell responses were assessed using Wilcoxon‐Rank sum testing. Spearman rank correlation coefficients were calculated for overall and subclass T‐cell responses after stimulation with antibody responses, and with symptom score (both as continuous variables).

## 3 RESULTS

### 3.1 Description of cohort

Of 431 individuals taking part in the home visit study, 384 had also participated sufficiently in the ZCS to allow the calculation of a symptom score.^6^ Participation in the current study is outlined in Supporting Information: Figure 1. Thirty‐four individuals had a symptom score predictive of COVID‐19 (“symptom‐positive”). Twelve of 34 could not be revisited (moved out of defined range, declined further involvement, did not respond to contact, or were symptomatic for COVID‐19 at time of planned repeat visit, precluding research team attendance). Of the remaining 22 in this group, 2 of the 15 who were IgG Spike antibody‐negative at the first home visit had seroconverted on reassessment. A further two symptomatic but initially antibody‐negative participants were excluded as repeat testing yielded inconsistent antibody results (Supporting Information: Figure 1).

Twelve asymptomatic participants were positive for both IgG Spike and Nucleocapsid antibodies. Six could not be re‐visited for one of the above reasons. Six asymptomatic antibody‐negative individuals were chosen as controls, based on proximity to other study participants (to minimize travel by the research team); however, one proved symptom‐positive on visiting and was reclassified.

Thus, the final numbers in the symptomatic group were 12 antibody‐negative and 9 antibody‐positive individuals, and in the asymptomatic group 5 antibody‐negative and 6 antibody‐positive individuals (Supporting Information: Figure 1). Demographic information on these participants within these final groupings is shown in Table 1. In symptomatic individuals, the median time from symptom onset to PBMC collection and repeat serology was 123 days (IQR: 111−130 days).

**Table 1:** Demographic data of participants, by grouping

	Total cohort	Ab+ S+	Ab+ S−	Ab− S+	Ab− S−
Total *n*	32	9	6	12	5
Age, mean (SD)	44.2 (13.4)	44.6 (9.0)	46.2 (19.5)	40.8 (12.3)	49.2 (16.2)
Sex (% female)	87	67	100	92	100

Abbreviations: Ab+, antibody‐positive; Ab−, antibody‐negative; S+, symptom‐positive; S−, symptom‐negative; SD, standard deviation.

### 3.2 T‐cell responses versus symptom scores

There was no association between T‐cell responses to SARS‐CoV‐2 peptides and either binary symptom status (Table 2; *χ* ^2^: 0.40, *p* = 0.529) or correlation with actual symptom score (*p* > 0.05 for all analyses; Table 3, Figure 1F). This was true for both T Helper and T Regulatory cells, as well as T Helper cell subsets inferred by expression of chemokine receptors (data not shown).

**Table 2:** Relationship between T‐cell responses, IgG Spike antibody status, and symptom status

	Combined T Helper + T Regulatory responses		
	Positive	Negative	Total
IgG Spike antibody status			
Positive	14	1	15
Negative	0	17	17
Total	14	18	32
Symptom status			
Positive	9	12	21
Negative	5	6	11
Total	14	18	32

**Table 3:** Correlation of quantitative T‐cell responses (overall and by subtype) after stimulation with SARS‐CoV‐2 peptides, with IgG Spike antibody fold change, and ZCS app symptom score

	Correlation with Ab response		Correlation to symptoms	
	Spearman's *ρ*	*p* value	Spearman's *ρ*	*p* value
T Helper response				
All SARS‐CoV‐2 peptides	0.83	<0.0001	0.16	0.39
S1 subunit	0.74	<0.0001	−0.04	0.83
S2 subunit	0.71	<0.0001	0.27	0.14
M + N subunits	0.83	<0.0001	0.10	0.6023
HA + INF	0.02	0.91	0.55	0.0018
SEB	0.17	0.37	0.39	0.0315
T Regulatory response				
All SARS‐CoV‐2 peptides	0.63	0.0001	0.22	0.23
S1 subunit	0.48	0.006	0.02	0.92
S2 subunit	0.49	0.005	0.31	0.95
M + N subunits	0.68	<0.0001	−0.06	0.76
HA + INF	−0.11	0.54	0.46	0.0096
SEB	0.05	0.79	0.26	0.16
Combined T‐cell response				
All SARS‐CoV‐2 peptides	0.79	<0.0001	0.17	0.35

Abbreviations: HA + INF, Infrarix Hexa‐vaccine and Influvac antigens; M + N subunits, matrix and neucleocapsid protein complex; S1, Spike Protein subunit 1; S2, Spike Protein subunit 2; SEB, superantigen enterotoxin B.

Figure 1T‐cell responses according to antibody and symptom status. (A) ROC curve for total T‐cell response compared to IgG S response. The area under curve: 0.85 (*p* = 0.0013). Star at optimal threshold (Increase of 0.22%, sensitivity 76.9%, specificity 80%). (B). Dot plot comparing the increase in Total T‐cell responses after stimulation with SARS‐CoV‐2 antigens, according to participant categories. (C) Frequency of activated T Helper cells for each participant, with bars subdivided into S‐1, S‐2, and M + N pools. (D) Frequency of activated T regulatory cells for each participant with bars subdivided into S‐1, S‐2, and M + N pools. (E) Total T‐cell responses plotted against IgG Spike antibody titer. Horizontal line (at *x* = 4) represents the threshold for defining IgG Spike response as positive.^13^ Vertical line (at *y* = 0.22) represents the threshold for defining a positive T‐cell response derived from our ROC analyses. (F) Total T‐cell response graphed against ZCS symptom tracker score. Horizontal line (at *x* = 0.5) represents cut‐off for defining individuals as symptomatic or asymptomatic.^12^ Vertical line (at *y* = 0.22) represents the threshold for defining a positive T‐cell response, derived from ROC analyses. AB, antibody; M + N, matrix and neucleocapsid proteins; ROC, receiver operating characteristic; S‐1, Spike protein subunit 1; S‐2, Spike protein subunit 2; Symp, Symptom.

**Figure:**
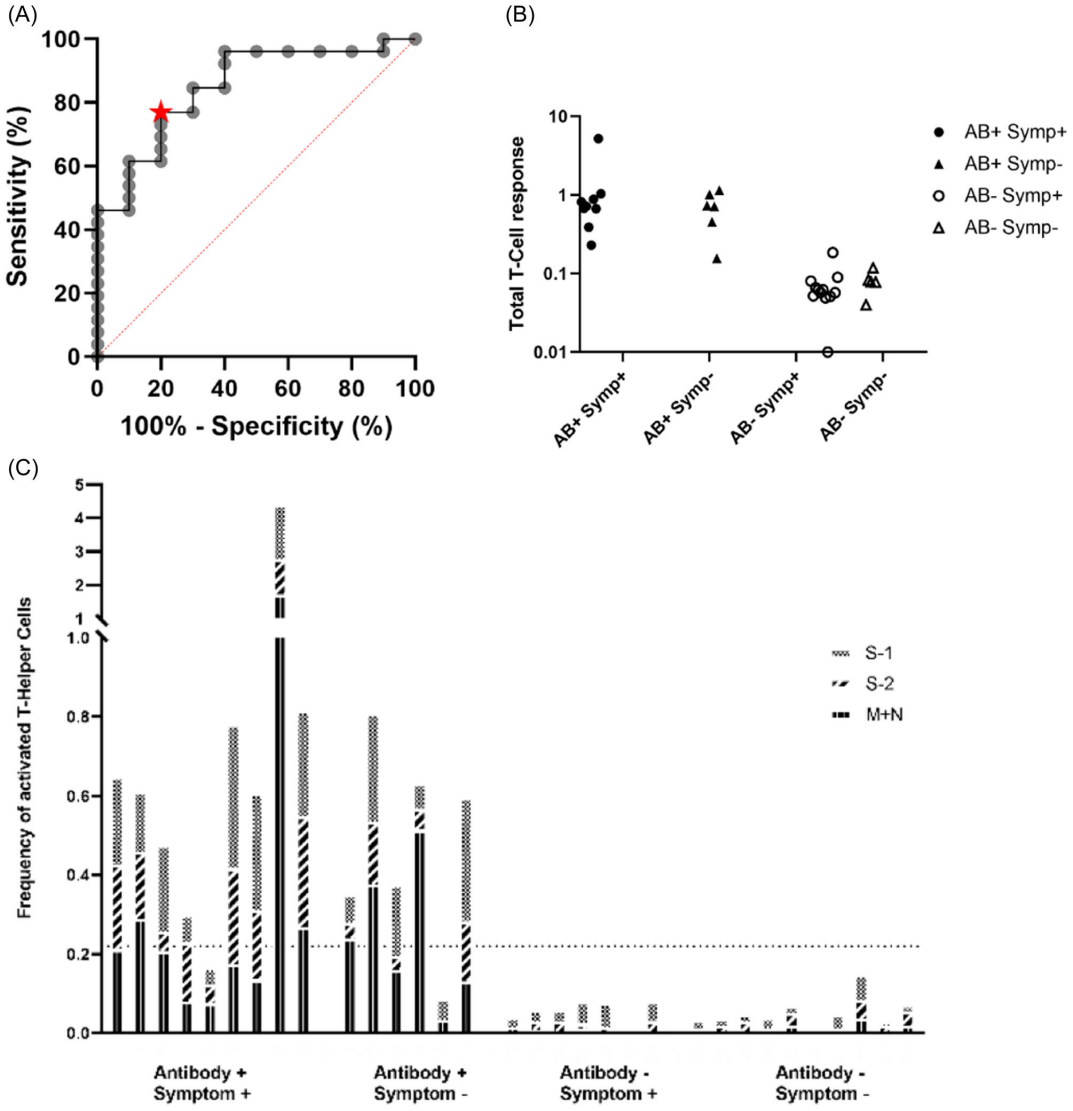

**Figure:**
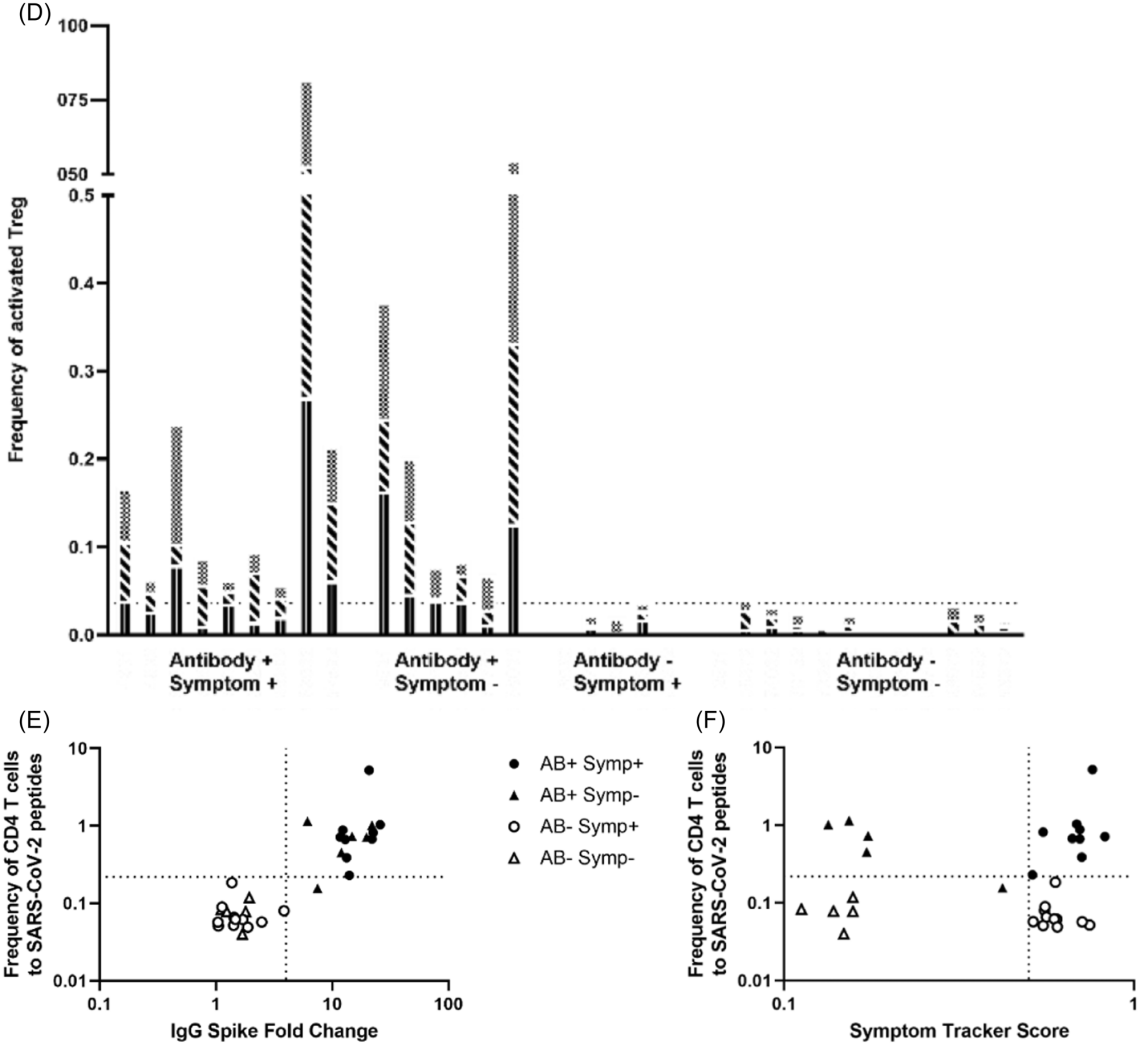

### 3.3 T‐cell responses and antibody status

Fourteen of 15 antibody‐positive individuals demonstrated a clear T‐cell response (*χ* ^2^ = 28.2, *p* < 0.001). In contrast, none of the 17 antibody‐negative participants showed a T‐cell response to antigen pools spanning Matrix, Nucleocapsid, and the S1 and S2 domains of Spike (categorical data shown in Table 2; qualitative T‐cell responses shown in Figure 1B−D). There were no further differences when examining subsets of T Effector or T Regulatory cells (Supporting Information: Figure 2).

### 3.4 Correlations between T‐cell responses and IgG Spike antibody titer

IgG Spike antibody titer correlated strongly with T‐cell responses to all SARS‐CoV‐2 antigen pools, considered as T‐cell responses overall (*ρ* = 0.79, *p* < 0.0001; Figure 1E) and as T helper and T regulatory responses individually (correlation of IgG Spike antibody titer with T helper responses: *ρ* = 0.83, *p* < 0.0001; and with T regulatory responses: *ρ* = 0.63, *p* = 0.0001, Table 3).

### 3.5 Associations with control antigens

There was no correlation between IgG Spike antibody level and T‐cell responses (overall or by subtype) to control antigens (HA + INF and SEB). Symptom score correlated with both T helper and T regulatory responses to HA + INF antigen stimulation (Table 3); however, this was only in those who were also IgG Spike antibody‐positive (*ρ* = 0.81, *p* = 0.0005) and not seen in those without IgG Spike antibodies (*ρ* = 0.33, *p* = 0.20).

## 4 DISCUSSION

Here we have shown a strong association and correlation between IgG Spike antibody responses and T‐cell responses to SARS‐CoV‐2 peptides, and importantly, symptomatic cases without spike antibodies after a wave of wild‐type infection (spring 2020) had no evidence of T‐cell memory of SARS‐CoV‐2 infection either.

Currently, the main use of IgG Spike antibody testing is to assess for the previous infection and/or vaccine immunogenicity. The reintroduction of restrictions in the availability of RT‐PCR means that future research will rely heavily on retrospective case identification of participants from patients with symptoms suggestive of COVID‐19. This may be particularly important in assessing participants with “Long COVID” and Post COVID‐19 Syndrome. Our data would suggest that testing of T‐cell responses is unlikely to add to the information gained from antibody testing in the first few months after infection. This is broadly concordant with findings in other groups, although without detailed information on symptoms.^17^ And although some others have found T cell responses in IgG Spike negative participants (without symptom data), this may have reflected cross‐reactivity from other seasonal corona viruses,^18^, ^19^ as reactivity was also noted in subjects without exposure to SARS‐CoV‐2 infection. In contrast, we used pre‐pandemic samples in defining response thresholds to SARS‐CoV‐2 peptides, and did not found cross‐reactivity.

The correlation of IgG‐S antibody titers was higher for T helper responses compared with T regulatory cells, as expected given the role of T helper cells in the generation of B‐cell antibody responses. However, a strong correlation was observed between T helper and T regulatory responses.

In defining symptomatic groups, we used a validated algorithm for predicting COVID‐19,^12^ although the data from this small cohort raise some questions about the robustness of this algorithm. Although IgG Spike antibody levels decline over time after natural infection, individuals were assessed on two occasions; categorization in this study was based on the contemporaneous collection of PBMCs and serology. Our laboratory methods included externally validated and published antibody testing methodology,^13^ and we parsed T helper and T regulatory cell responses using previously published methodologies.^14^, ^20^

Although our sample size is small (in part due to travel restrictions) and predominantly female (reflecting the whole TwinsUK cohort), it is not dissimilar to other studies.^17^, ^18^, ^19^ Although males are more severely affected by acute COVID‐19 although there is no a priori reason to suspect concordance between IgG‐Spike antibody status and T‐cell response would differ by gender. Although we did not assess for an isolated CD8^+^ response, it has been demonstrated elsewhere that CD8^+^ responses and CD4^+^ responses to SARS‐CoV‐2 are strongly associated.^21^ The close relationship we have observed between IgG Spike antibody titer and T‐cell reactivity may differ at later time points post infection, noting that T‐cell responses after SARS‐CoV‐1 infection can be extremely long‐lasting.^9^ While it would also be interesting to assess longitudinal patterns of IgG Spike and T‐cell responses to SARS‐CoV‐2 infection, our cohort, like many others in high‐income countries, is now vaccinated, and the overlap between responses to natural infection and vaccination precludes further analysis. Finally, at the start of our home visit study,^6^ community RT‐PCR testing was not routinely available in the United Kingdom: thus, we would be unable to detect infected individuals (i.e., RT‐PCR‐positive for SARS‐CoV‐2) who failed to mount either a sustained B and/or T‐cell response.

In conclusion, we have demonstrated a strong correlation between IgG Spike antibody response to SARS‐CoV‐2 infection and T‐cell reactivity (both T helper and T regulatory cells) against SARS‐CoV‐2‐derived peptides. Our study suggests that IgG Spike antibodies are a sufficient indication of recent infection, but as antibody titers decline over time, future research may be warranted to investigate the value of T‐cell responses in confirming historic SARS‐CoV‐2 infection. This may be of particular importance now that RT‐PCR testing during acute infection is becoming ever more restricted.

## AUTHOR CONTRIBUTIONS

Michael H. Malim, Katie J. Doores, Emma L. Duncan, Matthew A. Brown, Deborah Hart, Tim D. Spector, Claire J. Steves, and Timothy Tree conceptualized the study. Eleni Christakou, Timothy Tree, Ffion Harris, Yasaman Shahrabi, Emily Pollock, Muntaha Wadud, and Katie J. Doores performed the experiments. Marc F. Österdahl performed the statistical analysis and drafted the manuscript with Emma L. Duncan, Timothy Tree, and Katie J. Doores. All authors have contributed to revising the manuscript and oversight of its final form.

## CONFLICT OF INTEREST

T. D. S. is a shareholder and cofounder of ZOE Global Ltd., and has received payment for scientific consultancy services to ZOE Global Ltd. No other authors have competing interests to declare.

## ETHICS STATEMENT

The TwinsUK study was approved by NHS London—London‐Westminster Research Ethics Committee (REC reference EC04/015), and Guy's and St Thomas' NHS Foundation Trust Research and Development (R&D). The TwinsUK Biobank was approved by NHS North West—Liverpool East Research Ethics Committee (Reference: 17/NW/0187), IRAS ID 258513. All participants provided written, informed consent. The ZCS was approved by KCL Ethics Committee (REMAS ID 18210, review reference LRS‐19/20‐18210). Upon registration, all subscribers consented for their data to be available for COVID‐19 research. Participant samples for the ROC analysis were originally approved for use by the London‐Bromley Research Ethics Committee (Reference: 08/H0805/14), with an extension permitted under the extended Control of Patient Information (COPI) notice 2020/21 and specific approval from the Committee Chair for this study. All experiments were performed in compliance with local and national regulatory guidelines, and the Declaration of Helsinki.

## Supporting information

Supplementary information.

## Data Availability

To comply with informed participant consent, which stipulated raw anonymized individual‐level data would only be available upon request for research purposes, data collected using the COVID Symptom Study smartphone application are being shared with other researchers through the UK National Health Service‐funded Health Data Research UK (HDRUK) and Secure Anonymised Information Linkage consortium, housed in the UK Secure Research Platform (Swansea, UK, https://web.www.healthdatagateway.org/dataset/fddcb382-3051-4394-8436-b92295f14259). Data relating to TwinsUK participants should also be discussed with the Department of Twins Research and Epidemiology, King's College London. Further information can also be found on the TwinsUK website (www.twinsuk.ac.uk/data-access).
