# Supplementary material for: Concordance of B‐ and T‐cell responses to SARS‐CoV‐2 infection, irrespective of symptoms suggestive of COVID‐19
Source: J Med Virol. 2022 Jul 30;94(11):5217–24. doi: 10.1002/jmv.28016 (PMC9349709; doi:10.1002/jmv.28016)
Supplement: Supplementary file 1 — Supplementary information. [file JMV-94-5217-s001.pdf]

Supplementary Methods

Humoral assays

Spike and nucleocapsid protein were expressed as previously described[13]. All sera were heat-inactivated at 56°C for 30 mins before use. High-binding ELISA plates (Corning, 3690) were coated with antigen (Spike or N) at 3 µg/mL (25 µL per well) in PBS, either overnight at 4°C or 2 hr at 37°C. Wells were washed with PBS-T (PBS with 0.05% Tween-20) and then blocked with 100 µL 5% milk in PBS-T for 1 hr at room temperature. Wells were emptied and sera (diluted at 1:50 in milk) added and incubated for 2 hr at room temperature. Control reagents included CR3009 (2 µg/mL), CR3022 (0.2 µg/mL), negative control plasma (1:25 dilution), positive control plasma (1:50) and blank wells. Wells were washed with PBS-T. Secondary antibody was added and incubated for 1 hr at room temperature. IgM was detected using Goat-anti-human-IgM-HRP (1:1,000) (Sigma: A6907) and IgG was detected using Goat-anti-human-Fc-AP (1:1,000) (Jackson: 109-055-098-JIR). Wells were washed with PBS-T and either AP substrate (Sigma) was added and read at 405 nm (AP) or 1-step TMB substrate (Thermo Scientific) was added and quenched with 0.5 M H_2S0_4 before reading at 450 nm (HRP)

PBMC stimulation with SARS-CoV-2 overlapping peptide pools

PBMC were stimulated with pools of overlapping peptides spanning the whole sequence of the SARS-CoV-2 Matrix (M) and Nucleocapsid (N) proteins (Peptivator peptide pools, Miltenyi) and two pools spanning the S1 and S2 domains of the SARS-CoV-2 spike protein (Peptivator_Prot_S1 Miltenyi and PepMix SARS-CoV-2 vial 2, JPT Peptide Technologies). These peptide pools can stimulate both MHC-I and MHC-II restricted T cells without HLA bias[21]. Response to the S1 protein subunit is most comparable with IgG-S antibody testing. All peptides were used at a final concentration of 0.33 ng/µl. Superantigen Enterotoxin B (SEB) at 100ng/mL (Sigma Aldrich) was used as a positive control; Infanrix, a hexa-vaccine (GlaxoSmithKline) and Influvac, an Influenza surface antigen vaccine (Abbott Biologicals) were combined (HA + INF) and used to examine anamnestic responses induced by

vaccination or infection. Peptide diluent (DMSO) was used as a negative control. PBMC (1-2x106 /stimuli) were incubated for 18h at 37°C in 48-well plates in X-Vivo media (Lonza) supplemented with 5% human AB serum (Sigma) and 0.4µg/mL anti-CD40 antibody (BioXcell).

Flow Cytometry

Following incubation, PBMC were stained with a live/dead cell marker (LIVE/DEAD™ Fixable Near-IR Dead Cell Stain Kit - Invitrogen) and cell surface markers (Supplementary Table 1). Samples were acquired on a LSRFortessa Flow Analyser (BD Biosciences) and analysed using the software FlowJo (TreeStar Inc., version 10.7.2). As previously described, we defined activated conventional helper T cells based on the expression of CD69 and CD40L (CD154) and activated regulatory T cells based on upregulation of 4-1BB (CD137) and GARP in CD40L negative cells[14–16]. We also further defined T-Cell subsets based on chemokine receptor expression (CXCR3, CCR6, CCR4 CXCR6 and CXCR5), and on memory status (CD45RA, CCR7), for presentation in supplementary data (Supplementary Figure 2+3).

Antigen-specific T cell responses were described as the frequency of cells responding to each stimulus as a percentage of live total $CD4^+$ T cells following subtraction of unstimulated controls. Negative values were set to zero.

Stored PBMCs from anonymised healthy controls recruited pre-pandemic were used to define thresholds for T cell responses to SARS-CoV-2 peptides in ROC analysis. A threshold of 0.22% increase in frequency of live T cells responding to SARS-CoV-2 peptide pools was established as optimal (sensitivity 76.9%, specificity 80%) (Figure 1A).

Supplementary Table S1: Cell Surface markers used to identify T cell subsets

Cell Surface Marker	Clone/Fluorochrome	Manufacturer
**GARP**	Clone 7B11- APC	BioLegend
**GARP**	Clone G14D9-eFluor 660	eBioscience
**CD19**	Clone HIB19 -APC/Cy7	BioLegend
**CD137**	Clone 4B4-1 - BV421	BioLegend
**CD134/OX-40**	Clone Ber-ACT35 – BV605	BioLegend
**CD154**	Clone 24-31 - BV771	BioLegend
**CD69**	Clone FN50 - FITC	BioLegend
**CD14**	Clone HCD14 - APC/Cy7	BioLegend
**HLA-DR**	Clone L243 - PE-Dazzle 594	BioLegend
**CD4**	Clone RPA-T4 - BUV395	BD
**CD8**	Clone SK1 - BUV737	BD
**CD45RA**	Clone HI100 - BV785	BioLegend
**CCR7**	Clone 3D12 - APC-R700	BD
**CCR6**	Clone 11A9 - BV650	BD
**CXCR6**	Clone K041E5 - PE	BioLegend
**CXCR3**	Clone G025H7 - PE/Cy5	BD
**CXCR5**	Clone J252D4 - PE/Cy7	BioLegend

Supplementary Fig 1: Flowchart of enrolment into cohort

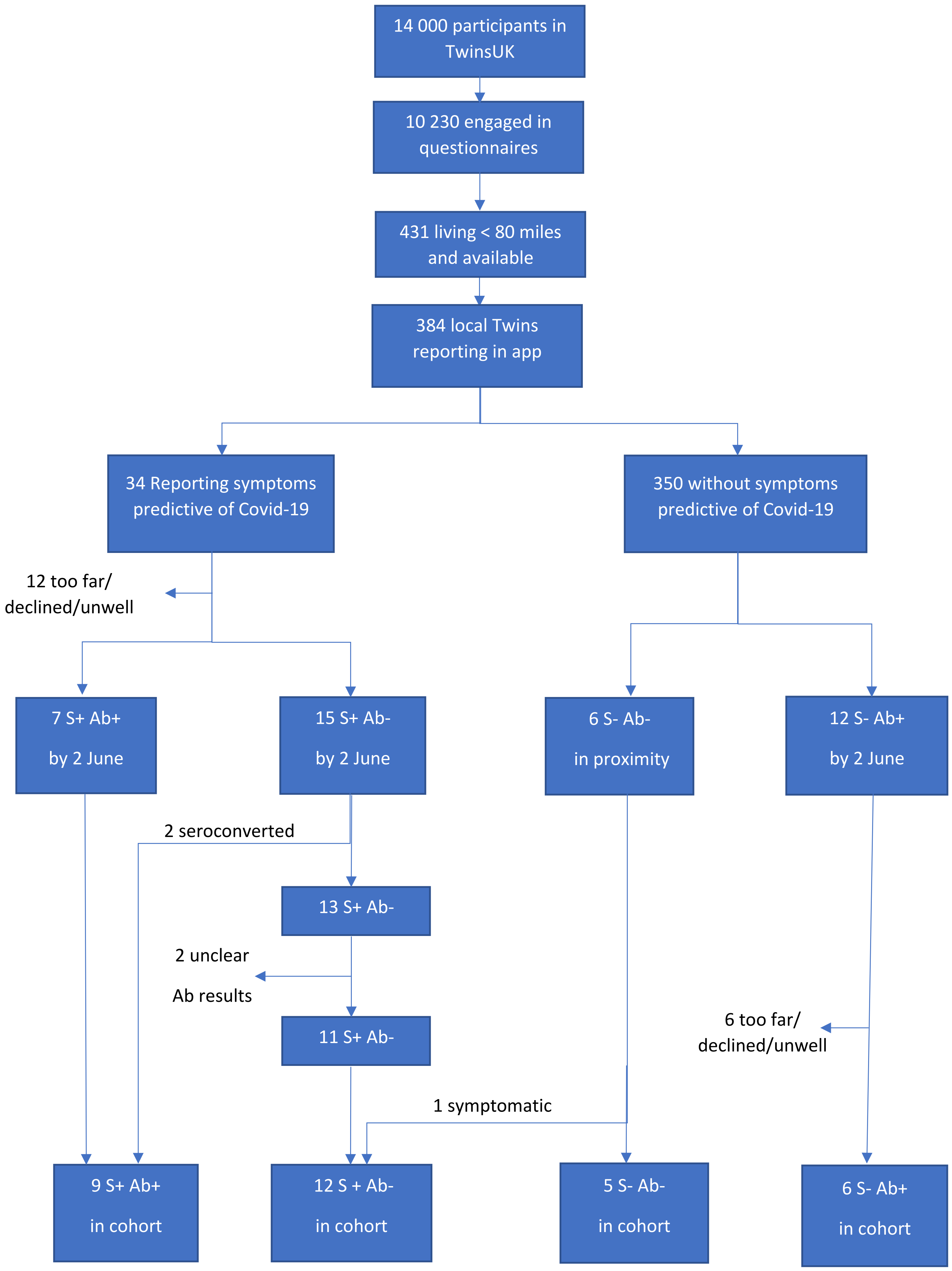

Supplementary Figure 2

A.

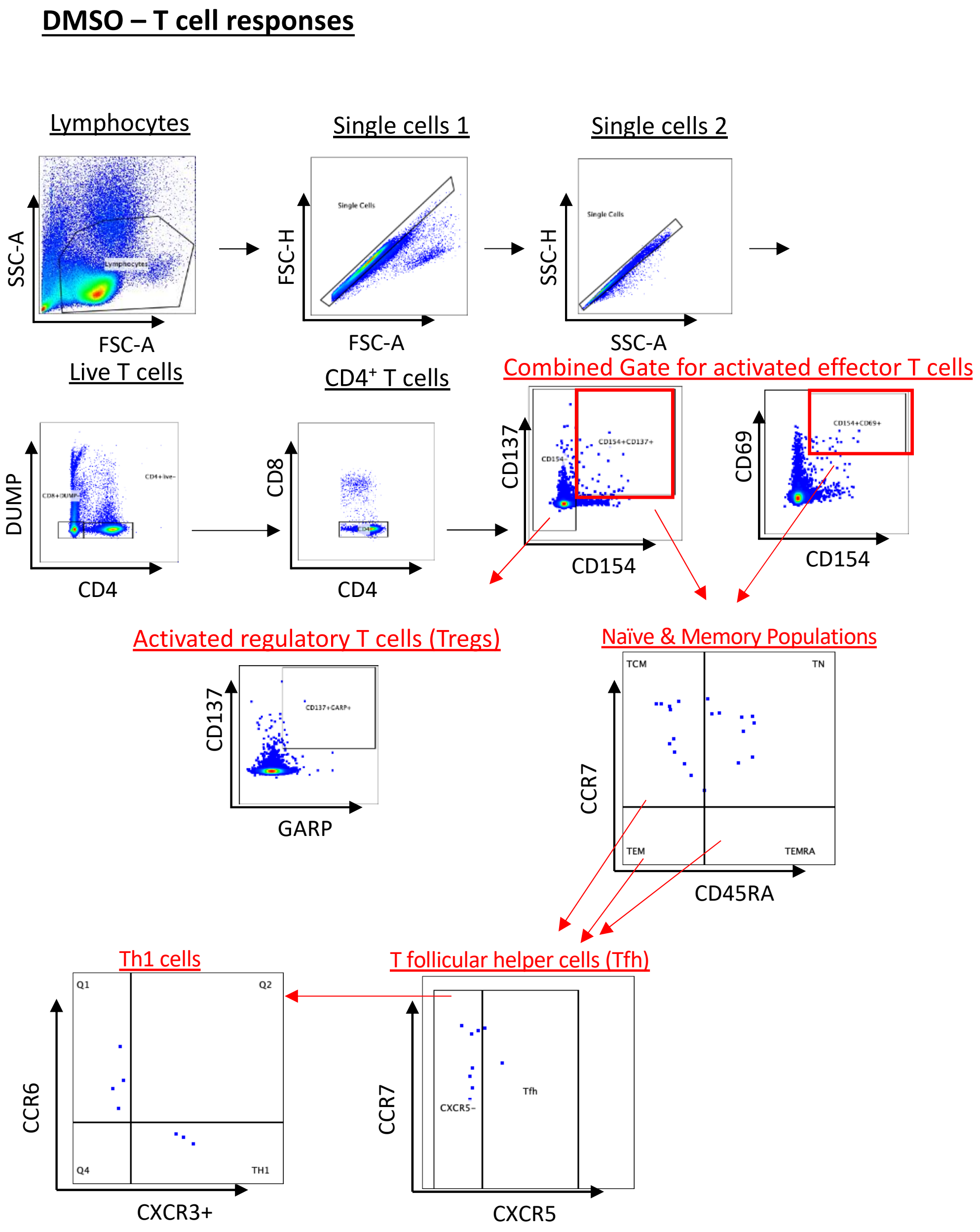

B.

Spike Protein Domain 1 - T cell responses

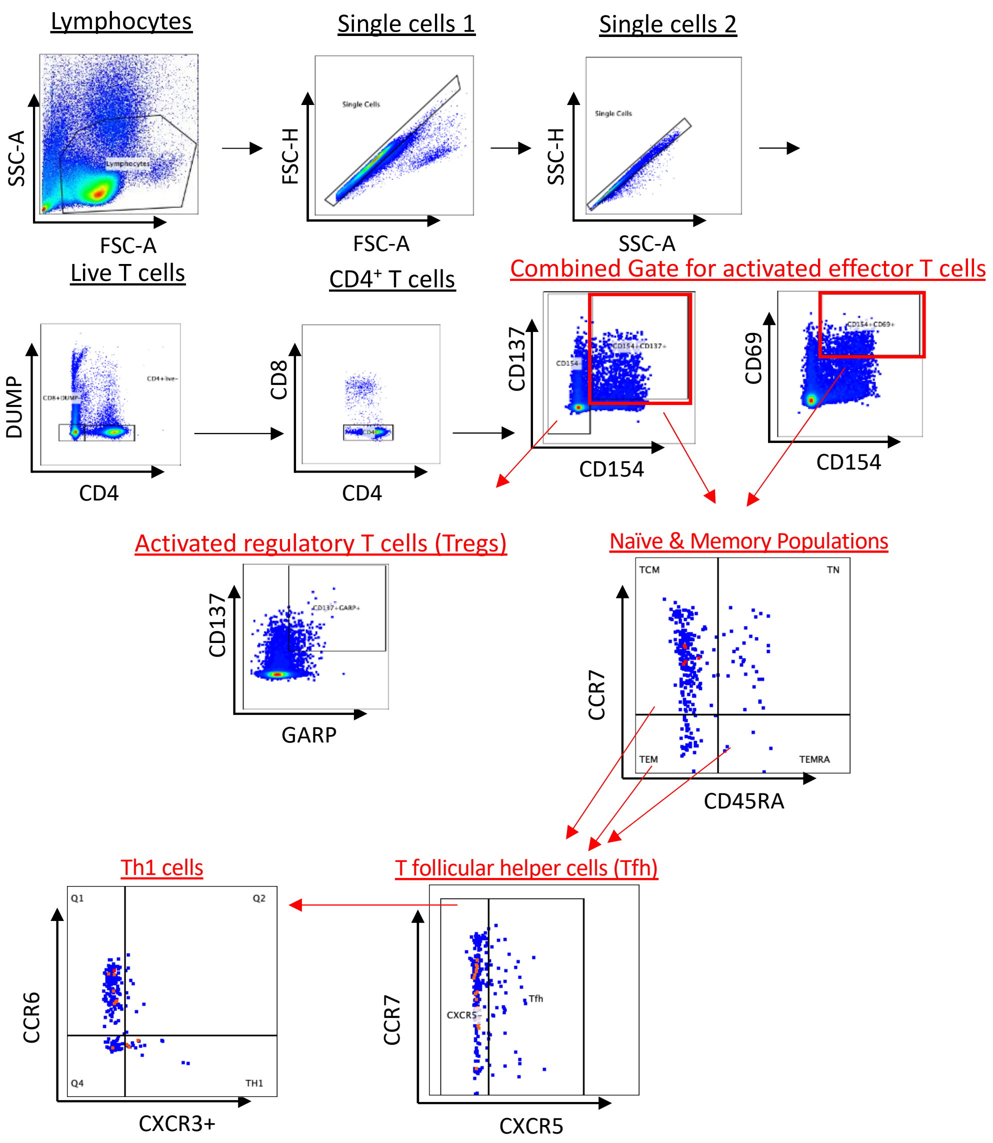

__Supplementary Figure 2:__ **Gating scheme for the identification of the antigen activated effector, regulatory T cells, memory and naïve populations and T helper 1 cells.** This figure represents an example of the gating strategy identifying the activated $CD4^+$ T cells by the used SARS-Cov-2 peptides. PBMCs were incubated with **A)** peptide diluent (DMSO) as a negative control and **B)** the domain 1 of the spike protein of SARS-CoV-2. Lymphocytes were gated based on SSC and FSC properties. From lymphocytes, doublet cells were excluded and live $CD4^+$ T cells were gated based on the CD4 expression and the DUMP channel. Further selection of $CD4^+$ T cells was achieved by excluding the $CD8^+$ T cells. The activated effector T cell responses were gated by combining the expression of CD154, CD137 and CD69. After the exclusion of $CD154^+$ cells, the regulatory T cell responses (Tregs) were gated based on the expression of CD137 and GARP. The identification of the memory (TCM, TEM, TEMRA) and naïve populations (TN) was achieved based on the expression of CCR7 and CD45RA from the activated T effector populations. Then by excluding the naïve T cells, T follicular helper cells (Tfh) were gated based on the expression of CCR7 and CXCR5. Finally, after the exclusion of the Tfh cells, T heper 1 cells (Th1) were gated based on the expression of CXCR3 and CCR6.

Supplementary Figure 3

3A

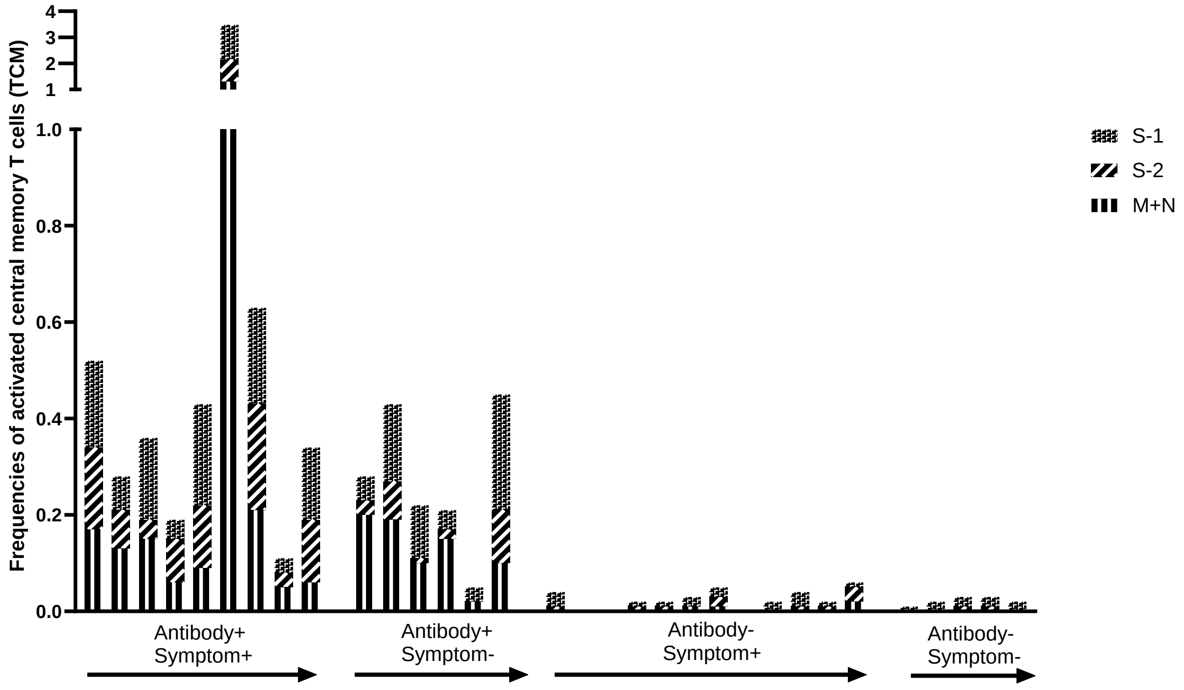

3B

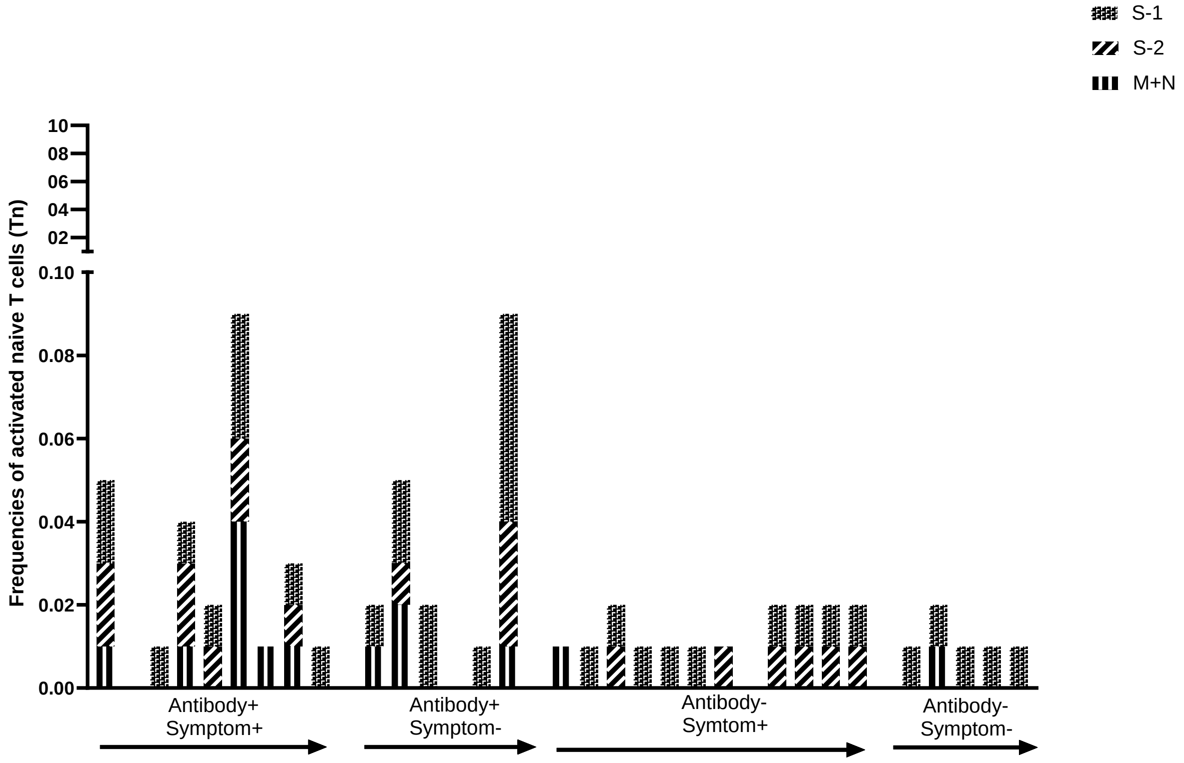

3C

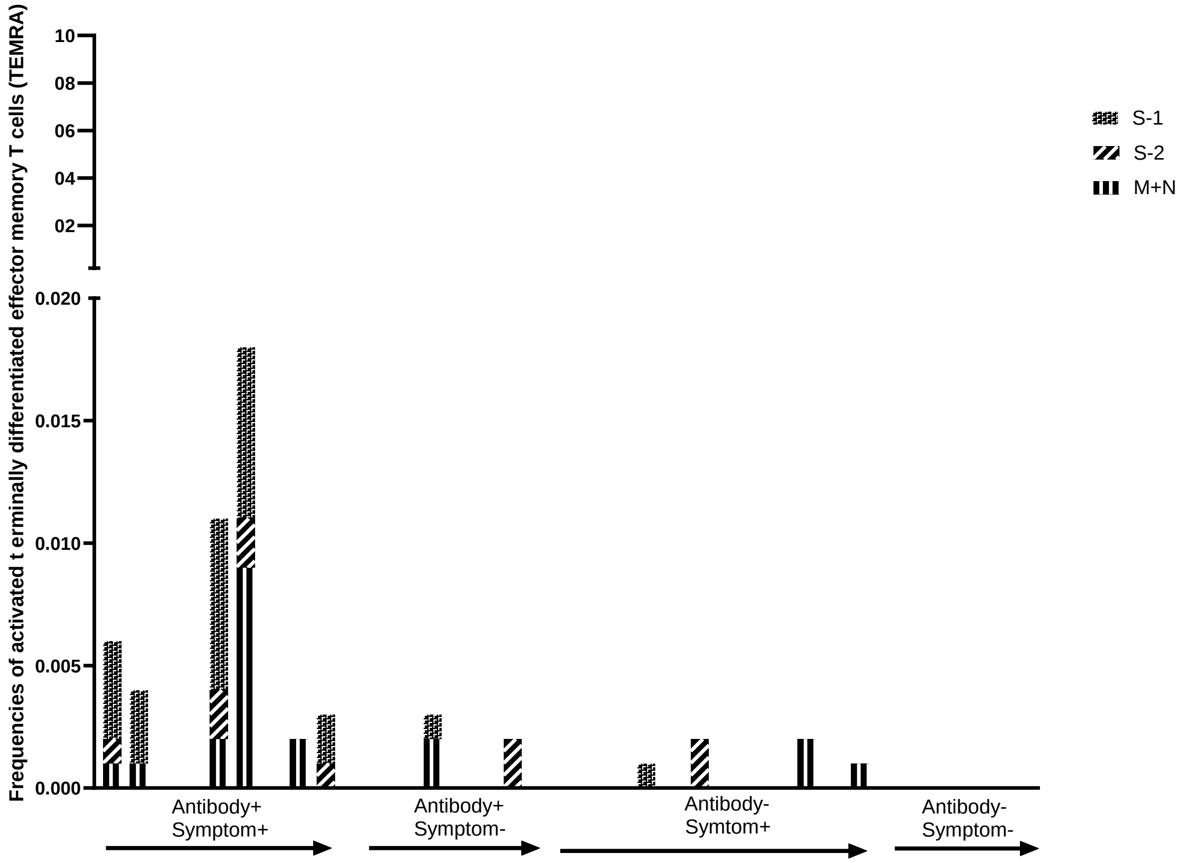

Frequencies of activated t erminally differentiated effector memory T cells (TEMRA)
10
08
06
04
02
0.020
0.015
0.010
0.005
0.000
S-1
S-2
M+N
Antibody+
Symptom+
Antibody+
Symptom-
Antibody-
Symtom+
Antibody-
Symptom-

3D

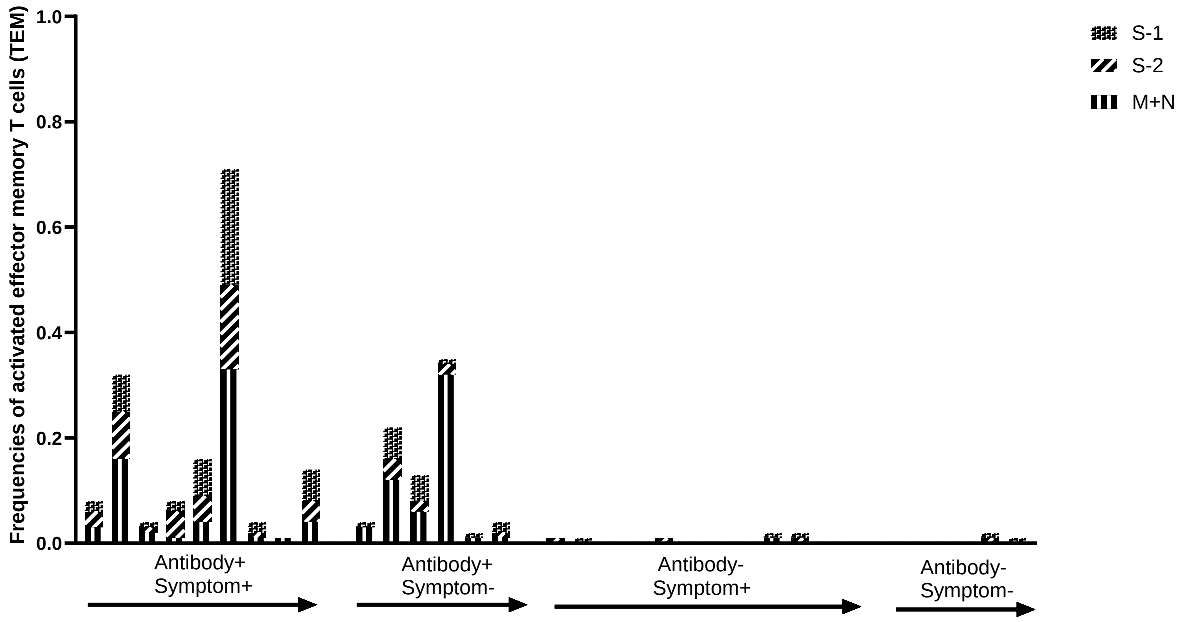

Frequencies of activated effector memory T cells (TEM)
1.0
0.8
0.6
0.4
0.2
0.0
S-1
S-2
M+N
Antibody+
Symptom+
Antibody+
Symptom-
Antibody-
Symptom+
Antibody-
Symptom-

3E

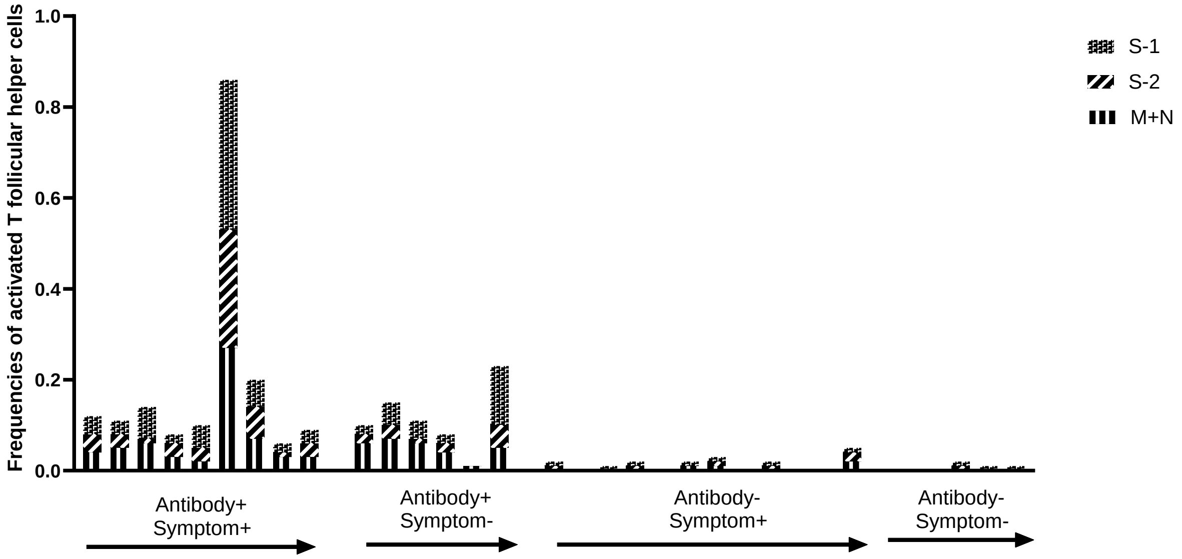

3F

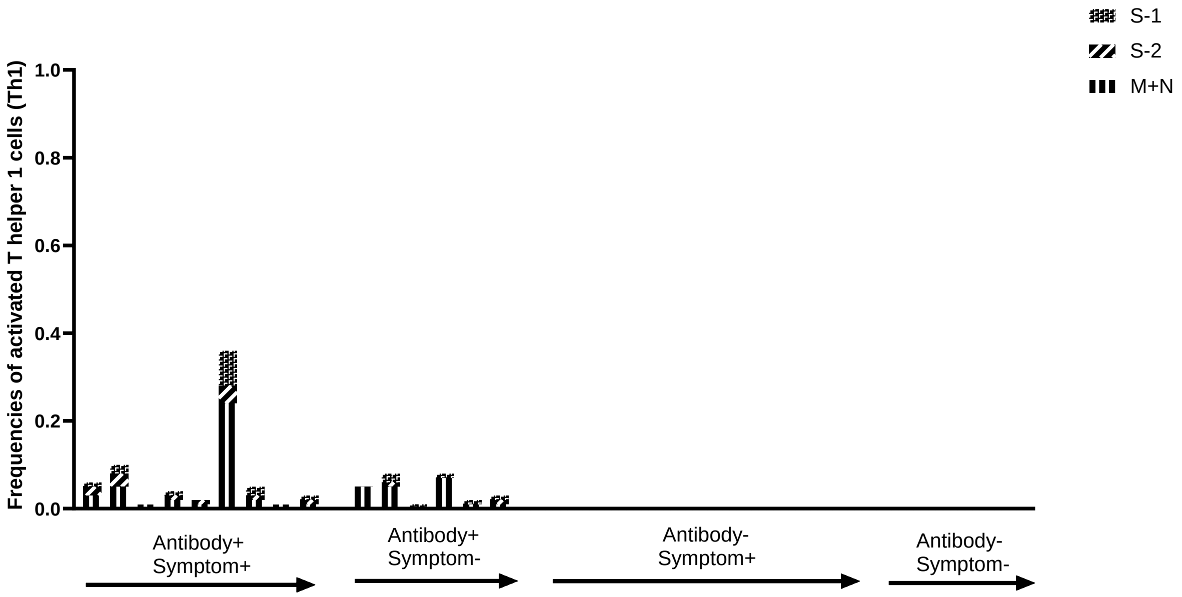

<u>Supplementary Figure 3:</u> T cell subset responses according to antibody and symptom status.

A) Frequency of activated central memory T cells (TCM) for each participant, with bars subdivided into S-1, S-2, and M+N pools.
B) Frequency of activated naive T cells (TN) for each participant, with bars subdivided into S-1, S-2, and M+N pools.
C) Frequency of activated terminally differentiated effector memory T cells (TEMRA) for each participant, with bars subdivided into S-1, S-2, and M+N pools.
D) Frequency of activated effector memory T cells (TEM) for each participant, with bars subdivided into S-1, S-2, and M+N pools.
E) Frequency of activated T follicular helper cells (Tfh) for each participant, with bars subdivided into S-1, S-2, and M+N pools.

F) Frequency of activated T helper 1 cells (Th1) for each participant, with bars subdivided into S-1, S-2, and M+N pools.

All T cell subsets are expressed as a frequency of total CD4 T cells.
